# The tip of the iceberg—The roles of long noncoding RNAs in acute myeloid leukemia

**DOI:** 10.1002/wrna.1796

**Published:** 2023-06-02

**Authors:** Patrick Connerty, Richard B. Lock

**Affiliations:** ^1^ Children's Cancer Institute, Lowy Cancer Research Centre UNSW Sydney Sydney New South Wales Australia; ^2^ School of Clinical Medicine UNSW Medicine & Health, UNSW Sydney Sydney New South Wales Australia; ^3^ University of New South Wales Centre for Childhood Cancer Research UNSW Sydney Sydney New South Wales Australia

**Keywords:** acute myeloid leukemia, cancer, long non‐coding RNAs, RNA biology

## Abstract

Long noncoding RNAs (lncRNAs) are traditionally defined as RNA transcripts longer than 200 nucleotides that have no protein coding potential. LncRNAs have been identified to be dysregulated in various types of cancer, including the deadly hematopoietic cancer—acute myeloid leukemia (AML). Currently, survival rates for AML have reached a plateau necessitating new therapeutic targets and biomarkers to improve treatment options and survival from the disease. Therefore, the identification of lncRNAs as novel biomarkers and therapeutic targets for AML has major benefits. In this review, we assess the key studies which have recently identified lncRNAs as important molecules in AML and summarize the current knowledge of lncRNAs in AML. We delve into examples of the specific roles of lncRNA action in AML such as driving proliferation, differentiation block and therapy resistance as well as their function as tumor suppressors and utility as biomarkers.

This article is categorized under:

RNA in Disease and Development > RNA in Disease

## Graphical abstract

The many roles and functions of long noncoding RNAs in acute myeloid leukemia.

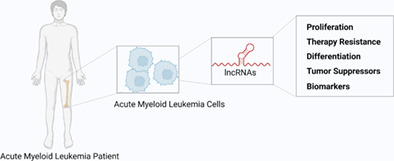

## 1 INTRODUCTION

Acute myeloid leukemia (AML) is one of the deadliest hematological malignancies with an overall survival (OS) rate of 30% in adults (Kantarjian et al., 2021). Currently, AML is treated with high‐intensity chemotherapy and bone marrow transplantation which cause severe side effects in patients. Moreover, the majority of patients will have their disease relapse, resulting in limited treatment options (Kantarjian et al., 2021). Consequently, there is a need to identify key genes which are crucial for initiation, maintenance, and therapy resistance of AML. Although the function of many protein‐coding genes has been extensively researched in AML, the majority of the human genome consists of noncoding transcripts, and these noncoding species have often been neglected.

Long noncoding RNAs (lncRNAs) are a class of noncoding RNA that were previously considered genetic “noise” because of their lack of protein‐coding ability. However, recent studies have found that noncoding RNAs play vital roles in human diseases and cancers (Qian et al., 2020). Key lncRNAs such as HOTAIR, NEAT1, and MALAT1 have now been discovered to drive the progression of many cancers (Qian et al., 2020). Aberrant expression of these oncogenic lncRNAs can promote cancer cell pathways such as unchecked proliferation, self‐renewal, migration, and therapy resistance. Conversely, many lncRNAs have been identified as tumor suppressors which regulate key apoptotic pathways, highlighting the varied roles of these genetic molecules (Guzel et al., 2020; Pang et al., 2019). While the molecular mechanisms of action for greater than 95% of lncRNAs are not clear to date, an increasing number of studies are identifying lncRNAs that have critical roles in cancer, including AML (Mishra et al., 2022). Over the last 3 years, other reviews have discussed the role of noncoding RNAs in hematological malignancies, however, they have focused on other noncoding RNA species such as microRNAs and circRNAs or have looked at lncRNAs in a pediatric setting (Bhattacharya & Gutti, 2022; Y. Liu et al., 2019; Neyazi et al., 2022). In this review, we analyze recent findings in the field of lncRNAs in AML with a focus on the specific mechanisms of lncRNAs in AML such as driving proliferation, differentiation block, therapy resistance as well as their function as tumor suppressors and utility as AML biomarkers.

## 2 ACUTE MYELOID LEUKEMIA

AML is one of the deadliest hematological malignancies, especially when compared to other common subtypes of acute leukemia. AML is characterized by uncontrolled proliferation and survival of undifferentiated myeloblasts. These immature myeloblasts accumulate in the bone marrow and peripheral blood causing anemia and eventually death. While the average age of incidence of AML is 70 years old, the disease affects both infants and elderly adults (Yi et al., 2020). Treatment options for AML include high‐intensity chemotherapy of cytarabine (Ara‐c) and daunorubicin, known as the 7 + 3 standard of care, as well as hematopoietic stem cell transplant (Yu et al., 2020). However, survival rates for this cancer remain low and treatment options remain limited for relapsed or therapy‐resistant disease (Rashidi et al., 2018).

One major limitation in finding effective treatments for AML is the extraordinary heterogeneity of the disease. Over the last few decades, a number of genetic abnormalities associated with AML have been discovered and clear risk stratification has been established for these cytogenetic subtypes (Bolouri et al., 2018; Hou & Tien, 2020). Furthermore, mutations in several genes, such as FLT3, IDH2, NPM1, and CEBPA, have also been identified to stratify risk (Pourrajab et al., 2020). It is now well accepted that different cytogenetic subgroups of AML have different gene expression profiles and respond differently to treatment. For example, KMT2A/MLL1‐rearrangements generally cause an aggressive disease with a poor prognosis while APML cases are considered to be of favorable outcome and respond positively to all‐trans retinoic acid (ATRA) based therapy (Pourrajab et al., 2020). Likewise, mutations in genes such as DNTM3a and IDH2 are far more common in adults than in pediatric AML patients (Schuback et al., 2013). As our understanding of the human genome has improved and an increasing number of cancer‐associated genes have been discovered, unsurprisingly, the nonprotein‐coding portion of our genome has also been recognized to show distinct expression profiles in cancer. These breakthroughs in the discovery of functional noncoding RNAs, especially in the case of lncRNAs, provide an opportunity to shift from the “protein‐centric” view of cancer biology and provides researchers and clinicians with a new list of molecular drivers, therapeutic targets, and opportunities for hard‐to‐treat cancers such as AML.

## 3 LONG NONCODING RNAs

In the last decade, the role of noncoding RNAs as key regulators in a wide range of cellular processes has become increasingly apparent (Yao et al., 2019). Over 95% of the human genome is noncoding and these noncoding RNA products are mainly classified by their size. RNA species that are <200 nucleotides (nt) are classified as short noncoding RNA and, accordingly, those >200 nt are classified as lncRNAs (Dahariya et al., 2019). However, these definitions based on length are arbitrary and have no indication of the function of the RNAs. In fact, a recent consensus from Mattick et al., has suggested a reclassification of long noncoding RNAs to be defined as >500 nt in length and that 50–500 nt noncoding RNA species should be defined as RNA Pol III or Pol V transcripts (Mattick et al., 2023).

lncRNAs are processed similarly to mRNAs in that they are transcribed by RNA polymerase II, they are often 5′‐capped and they undergo post‐transcriptional modifications such as splicing (Quinn & Chang, 2015). Accordingly, many lncRNAs do not have any biochemical distinction from mRNAs besides lacking a translated open reading frame (ORF). Unlike mRNAs, many lncRNAs are sometimes not polyadenylated and undergo special processing events such as 5′‐ and 3′‐bookending by processed small nucleolar RNAs, and cleavage by RNase P. Additionally, lncRNAs are as commonly localized in the nucleus as the cytoplasm. Furthermore, the expression profile of lncRNAs is highly disease specific, with lncRNA expression profiles between cancer and healthy cells differing greatly, even in the context of AML (de Goede et al., 2021; Farrar et al., 2023; Quinn & Chang, 2015).

These loose definitions of lncRNAs encompass a large variety of long noncoding molecules. Therefore, lncRNAs are often further classified by their location in the genome relative to protein‐coding genes.

### 3.1 Classification

LncRNAs can be classified into five main categories based on their relative location to protein‐coding genes. These classifications include intronic, intergenic, sense, antisense, and bidirectional lncRNAs. Intronic lncRNAs are restricted to protein‐coding gene introns and can either be independent unique transcripts or created from pre‐mRNA splicing mechanisms. Intergenic lncRNAs (lincRNAs) do not overlap with protein‐coding genes and are located in the stretches of intergenic space present in the human genome. Sense lncRNAs, as their name suggests, are transcribed from the sense strand of protein‐coding genes and therefore contain exons from protein‐coding genes. Anti‐sense lncRNAs, are the opposite of sense lncRNAs and are transcribed from the anti‐sense strand of protein‐coding genes. Bi‐directional lncRNAs are similar to anti‐sense lncRNAs but located within 1 kb of the transcriptional start site of a protein‐coding gene and do not overlap, or only partially overlap, with their paired protein‐coding gene. While these definitions help distinguish lncRNA species, it should be noted that these classifications are not predictive of lncRNA function (Yao et al., 2019).

Despite their name and these traditional classifications, researchers have now discovered a new class of lncRNAs that contain short ORFs that encode small peptides (Choi et al., 2019). Moreover, it has also been shown that these small peptides are the key drivers of molecular function, not the RNA species. This phenomenon has been documented in plants as early as 2002 but the study of novel lncRNA‐encoded small peptides in mammals is in its infancy (Xing et al., 2021). Furthermore, the role of lncRNA‐encoded small peptides in cancer and other disease is a relatively untouched field that is now revealing the importance of these peptides in disease suppression and function (Xing et al., 2021). Accordingly, there have not been any peptide‐coding lncRNAs identified in the context of AML, however, the discovery that some lncRNAs are in fact peptide‐coding and that these peptides are key regulators of biological pathways is testimony to the fact that there is a major research gap in the role and functions of lncRNAs.

## 4 lncRNA FUNCTION IN CANCER AND AML

**FIGURE 1:**
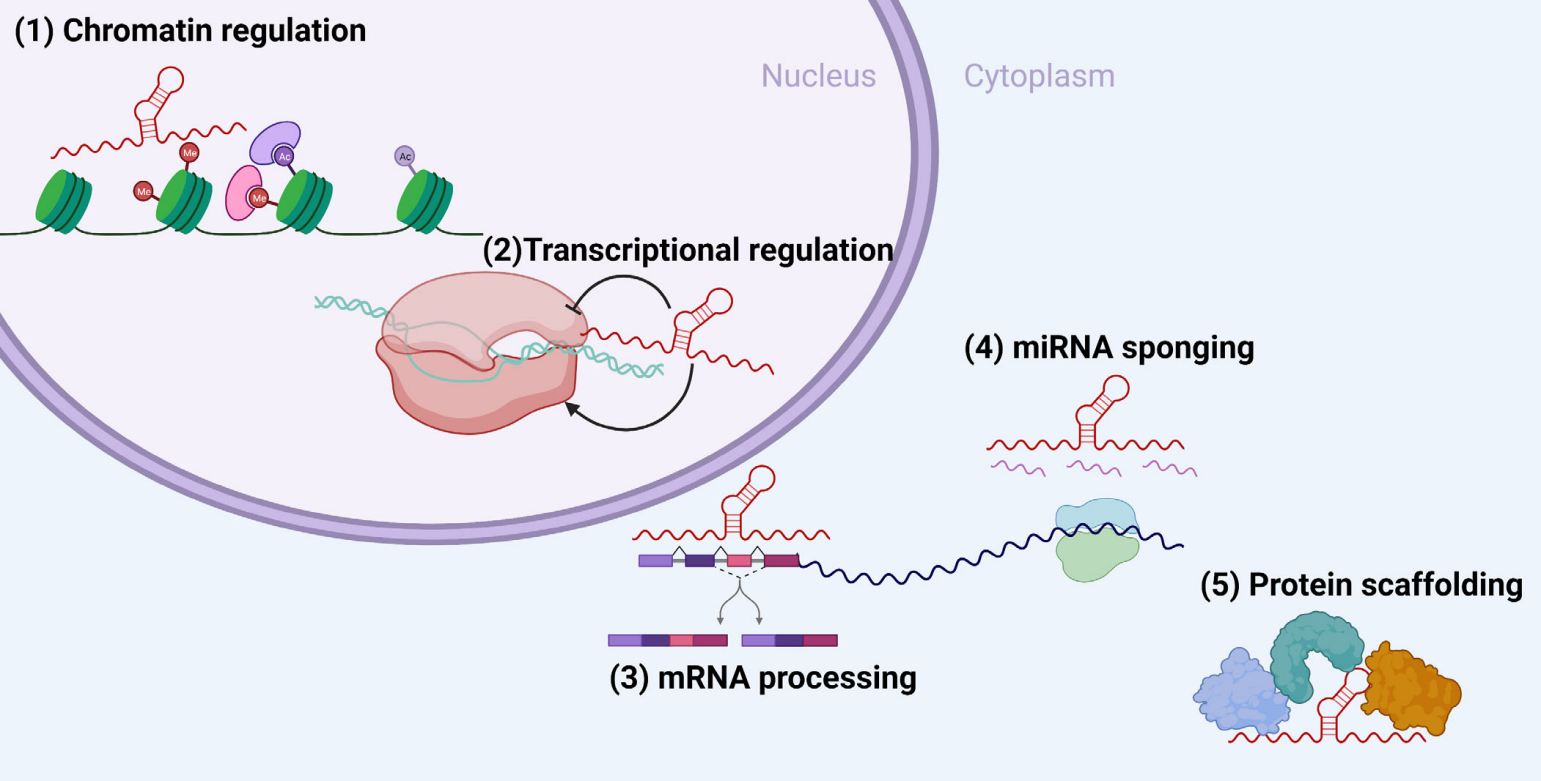
lncRNAs regulate cellular functions through a variety of mechanisms. lncRNAs regulate cellular processes through (1) regulating chromatin access, (2) promoting or inhibiting transcription of key genes, (3) mRNA processing and splicing, (4) sponging miRNAs from target mRNAs, and (5) acting as protein scaffolding RNAs to enable the formation of protein complexes.

Unlike proteins, lncRNAs with analogous functions often have no sequence homology; thus, the identification of the function of one lncRNA rarely informs the function of others (Kirk et al., 2018). There are several means by which lncRNAs can control cellular processes, and regulation by lncRNAs occurs at the beginning of transcription through to post‐translational processes. These methods include chromatin regulation, controlling transcriptional activation, guiding RNA processing, acting as miRNA sponges and protein scaffolds (Figure 1; Dahariya et al., 2019; Mattick et al., 2023; Yao et al., 2019). It is possible that these functions should be used to define lncRNAs rather than sequence length or genomic positions. However, these mechanisms are not mutually exclusive and the context‐specific role of lncRNAs means that a lncRNA can have two different functions in two different cell types. For example, the lncRNA MALAT1 has been reported as a miRNA‐sponging tumor suppressor in breast cancer but a transcription‐regulating oncogene in mantle cell lymphoma (Q. Chen et al., 2020). Through the mechanisms listed above lncRNAs tightly regulate important cellular processes and in the event of cancer lncRNA expression can be perturbed and result in the dysfunction of these crucial pathways.

Given the above, recent developments on the role of lncRNAs in cancer have identified them as key drivers of proliferation, differentiation, therapy resistance, tumor suppressors, and biomarkers in a range of cancers. The emerging role of lncRNAs in cancer presents an opportunity to identify novel oncogenes for AML. Recent studies have shown that lncRNAs are crucial in AML and that lncRNA expression profiles are specific to AML subtype (Connerty et al., 2021; Smith et al., 2019). However, though there are over 50,000 lncRNAs in the human genome, 95% have no described function (Maruyama & Yokota, 2020). Researchers have so far discovered an ever‐increasing number of lncRNAs to be physiologically important in AML through a variety of roles described below, thereby highlighting the importance of studying these molecules (Table 1).

**TABLE 1:** The cellular functions of lncRNAs in acute myeloid leukemia (AML).

lncRNA	Functions in AML	Targets	References
UCA1	Promote proliferation, Dox resistance	miR‐204/SIRT1 miR‐125/HIF‐1α	(Hughes et al., 2015; Y. Liang et al., 2020; Y. Zhang et al., 2018)
DUBR	Promote proliferation	miR‐142‐3p/FUS	(Yin et al., 2021)
ANRIL	Promote proliferation	AdipoR1 and miR‐34/HFAC1	(Sun et al., 2018; C. H. Wang et al., 2020)
HOXB‐AS3	Promote proliferation, inhibit differentiation	Ribosomal RNA transcription	(Papaioannou et al., 2019; Zhu et al., 2021)
MORRBID	Promote proliferation, inhibit apoptosis	BIM	(Cai et al., 2020)
HOTAIR	Promote proliferation, promote differentiation, and inhibit differentiation	HOXA5 and DNMT3B/EZH2/miR‐17‐5p and p21	(L. Chen, Hu, et al., 2020; Gao et al., 2018; Hu et al., 2021; L. Liang et al., 2021; S. L. Wang et al., 2019)
HOXA10‐AS	Promote proliferation	NF‐κB	(Al‐Kershi et al., 2019)
LINC01257	Promote proliferation	Unknown	(Connerty et al., 2021)
HOTTIP	Inhibit differentiation	HOXA genes and transcription factors (STAT4a, MYC, RUNX1, and DOT1L)	(Luo et al., 2019)
HOXBLINC	Inhibit differentiation	HOXA and HOXB cluster genes.	(Zhu et al., 2021)
LONA	Inhibit differentiation	THBS1 and ASB2	(Gourvest et al., 2021)
DANCR	Ara‐C resistance	miR‐874‐3p‐ATG16L1	(H. Zhang et al., 2021)
XIST	Dox resistance	MYC	(C. Wang et al., 2020)
HOTAIRM1	Dexamethasone resistance, Ara‐c resistance	ARHGAP18 and BCL2/β‐Catenin and c‐MYC	(L. Chen, Hu, et al., 2020; L. Liang et al., 2021)
LINC00152	Dox resistance	PARP1	(Cui et al., 2021)
GAS6‐AS2	Ara‐C resistance	GAS6/TAM pathway	(Bester et al., 2018)
NR‐104098	Tumor suppressor	EZH2	(Feng et al., 2020)
LOUP	Tumor suppressor	PU.1	(Trinh et al., 2021)
MAGI2‐AS3	Tumor suppressor	LRIG1	(L. Chen, Fan, et al., 2020)
NEAT1_1	Tumor suppressor	C/EBPβ, DVL‐2	(Yan et al., 2021)
MEG3	Tumor suppressor	DNTM3A/p53	(Lyu et al., 2017)

Abbreviations: Ara‐C, cytarabine; Dox, doxorubicin.

### 4.1 Proliferation

**FIGURE 2:**
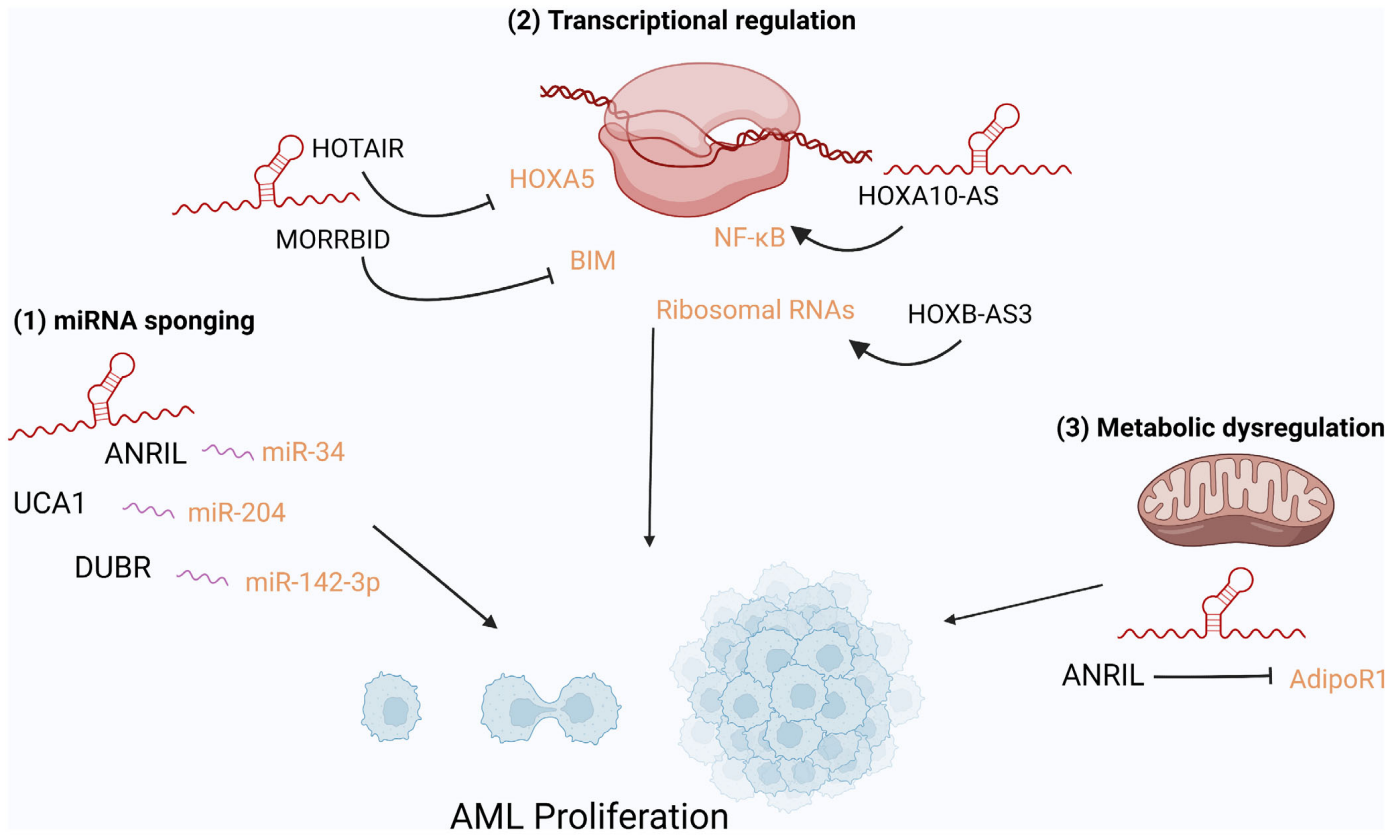
lncRNAs promote proliferation in AML. lncRNAs regulate AML proliferation through a variety of mechanisms such as (1) miRNA sponging of tumor suppressor miRNAs, (2) transcriptional regulation of pro‐ and anti‐apoptotic genes, and (3) metabolic dysregulation to promote rapid cell growth.

Sustaining proliferative signaling and evading growth suppressors are two of the hallmarks of cancer as defined by Hanahan (2022). The unchecked proliferation of cancer cells is definitive of the disease and many protein‐coding oncogenes have well‐established roles in dysregulating proliferative and growth suppressor pathways (Feitelson et al., 2015). Likewise, lncRNAs have also been found to drive proliferation in a range of cancers and this is also true in the context of AML. While the list of lncRNAs that drive proliferation in AML is small, new lncRNAs are constantly being discovered to promote AML cell growth through a variety of mechanisms (Figure 2).

For example, in 2015 the lncRNA UCA1 was discovered to be overexpressed in AML patient samples and promote proliferation of AML cell lines by repressing p27kip1 (Hughes et al., 2015). However, the exact mechanism by which UCA1 regulated p27kip1 and therefore cellular proliferation was not determined until 2020 when Liang et al demonstrated that UCA1 acts as a miRNA sponge for miR‐204 and thereby relieves the oncogene SIRT1 from miRNA silencing. SIRT1‐mediated downregulation of p27Kip1 is responsible for the unchecked growth of these cells (Y. Liang et al., 2020). Similarly, the lncRNA DUBR has been reported to promote cell proliferation in AML. Yin et al. (2021) demonstrated that high expression of DUBR was associated with poor outcome in AML and that siRNA‐mediated knockdown of DUBR suppressed the proliferation of AML cells. Using RNA pull‐down assay the authors determined that DUBR sponged miR‐142‐3p and thereby relieved the inhibition of the oncogenic FET protein, FUS.

lncRNAs have also been reported to cause the same phenotype through multiple roles even in the same context. For example, the lncRNA ANRIL, which is overexpressed in AML and other cancers, has been reported to promote AML cell proliferation through a variety of mechanisms. Sun et al. (2018) reported that ANRIL regulates proliferation and cell survival of the AML cell line MOLM13 through modulating leukemic cell metabolism. Their study found that high ANRIL expression enhances glucose uptake in AML cells by repressing the expression of Adiponectin receptor (AdipoR1), a key regulator of glucose metabolism. ANRIL was found to promote glucose uptake via dysregulating the AdiopR1/AMPK/SIRT1 signaling pathway. In contrast, Wang et al reported that ANRIL promotes cell proliferation in the HL‐60 cell line through sponging the tumor suppressor miR‐34 and thereby dysregulating miR‐34 targeting of the anti‐apoptotic protein HDAC1. These results demonstrate the multiple functions of lncRNAs and further emphasize the context specificity of lncRNA action (C. H. Wang et al., 2020).

Other lncRNAs have also been discovered to promote AML cell proliferation albeit through an alternative method than miRNA sponging. In a study by Papaioannou et al. (2019), HOXB‐AS3 was found to be highly expressed in patients with NPM1‐mutated AML and absent in healthy hematopoietic cells. HOXB‐AS3 knockdown reduced the proliferative capacity of NPM1‐mutant AML blasts in vitro and in vivo via relocating EBP1‐NPM1 complexes to the ribosomal DNA locus. Through this mechanism, HOXB‐AS3 promotes ribosomal RNA transcription and de novo protein synthesis, and through increasing protein production, HOXB‐AS3 enables leukemic blasts to rapidly proliferate. Likewise, the lncRNA MORRBID also regulates AML proliferation through transcriptional regulation. Expression of the lncRNA MORRBID is associated with FLT3^ITD^ and TET2 mutations and MORRBID was identified as a transcriptional repressor of BIM. Through repressing BIM, MORRBID is able to suppress the apoptotic pathways in murine and human AML blasts, resulting in unchecked proliferation. Accordingly, loss of MORRBID induces increased expression of BIM and promotes cell death, prolonging the survival of mice with MORBBID depleted AML (Cai et al., 2020).

Many lncRNAs which drive proliferation are not specific to AML, for example, the well‐characterized oncogenic lncRNA HOTAIR. HOTAIR, like in many cancers, is significantly overexpressed in leukemic compared to healthy haemopoietic cells. Wang et al, demonstrated that shRNA‐induced silencing of HOTAIR led to enhanced apoptosis and repressed proliferation of the AML cell line HL‐60. HOTAIR was found to methylate the promoter region of the tumor suppressor *HOXA5* via binding DNMT3B, and thereby repressing *HOXA5* expression (S. L. Wang et al., 2019). This function of HOTAIR is not unique to AML, however, and the same mechanism of action has been reported in breast cancer (Abba et al., 2015). Likewise, the lncRNA HOXA10‐AS has been identified to be oncogenic in glioma, gastric cancer and AML (Dong et al., 2018; S. Li et al., 2022). HOXA10‐AS is a stem cell‐specific lncRNA that drives proliferation in *KMT2A*‐rearranged AML. HOXA10‐AS is highly expressed in normal HSCs as well as in *KTM2A*‐rearranged and *NPM1* mutant AMLs. Overexpression of HOXA10‐AS in cell lines and *KMT2A*‐rearranged patient blasts resulted in increased proliferation through the induction of the NF‐κB pathway, whereas GapmeR‐ and CRISPR/Cas9‐induced knock out of HOXA10‐AS reduced proliferation and induced cell death (Al‐Kershi et al., 2019).

In contrast, some lncRNAs have been reported to be specific to AML. The lncRNA LINC01257 has been identified as an AML1‐ETO specific lncRNA. While LINC01257 was found to be high in AML1‐ETO patient samples and AML cell lines, its expression was low in healthy CD34^+^ cells and other AML subtypes. High expression of LINC01257 was associated with poor patient outcome, and siRNA‐induced knockdown reduced AML1‐ETO AML cell proliferation and induced apoptosis (Connerty et al., 2021). While the specific mechanism of LINC01257 action is still to be determined, the oncogenic role of this lncRNA has not been described in other cancer contexts.

Together, the studies listed here demonstrate the importance of lncRNAs in maintaining AML proliferation and thereby maintenance of the disease. Therefore, targeting lncRNAs to inhibit the unchecked proliferation of AML cells has significant potential as a therapeutic strategy. As many of the above studies demonstrated, lncRNA expression is often specific to AML cells, and thus lncRNA‐targeting therapies could have an advantage over traditional chemotherapies which indiscriminately target rapidly dividing cells.

### 4.2 Differentiation

**FIGURE 3:**
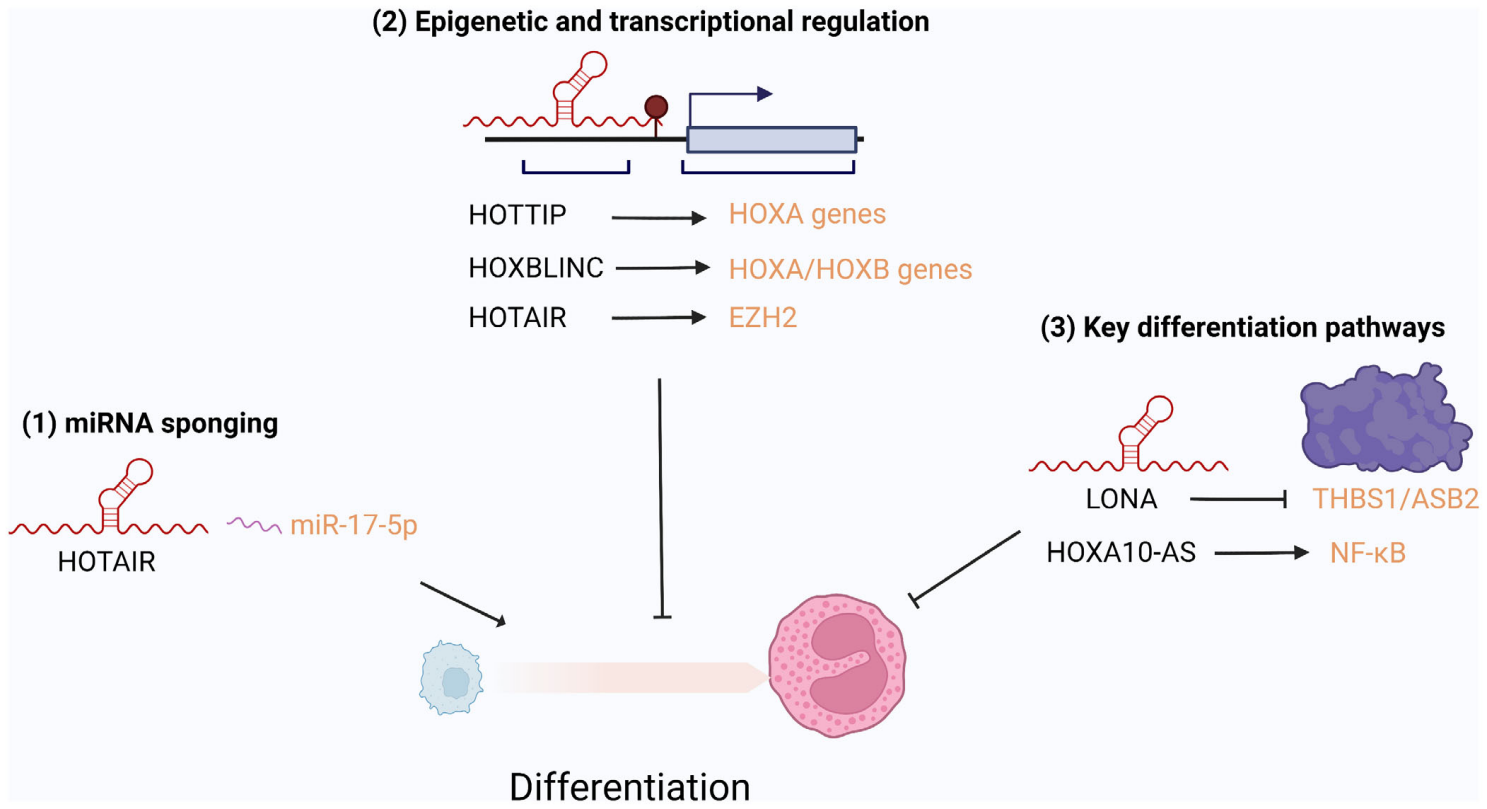
lncRNAs regulate differentiation in AML. lncRNAs can both inhibit or promote AML blast differentiation through a variety of mechanisms such as (1) miRNA sponging, (2) epigenetic regulation of genes that block differentiation and promoting, or (3) inhibiting key differentiation pathways.

A key feature of AML is the block of differentiation in myeloblasts to mature myeloid cells. This differentiation block and accumulation of immature myeloblasts is definitive of the disease. In normal hematopoiesis, myeloid cell differentiation is governed by a diverse network of epigenetic and transcriptional regulators (Schwarzer et al., 2017). In the case of AML, this differentiation process is halted through a variety of mechanisms such as chromosomal translocations and subsequent aberrant expression of transcription factors vital for differentiation events (Nemeth & Bodine, 2007; Rodrigues et al., 2021). An ever‐growing number of lncRNAs have now been identified to contribute to differentiation arrest and therefore are vital for the maintenance of the disease (Figure 3).

The lncRNA HOTTIP is one such lncRNA that halts the differentiation of myeloid cells in AML. In a recent study by Luo et al, HOTTIP was identified as overexpressed in AML patients compared to healthy controls. The authors determined that HOTTIP alters HOXA‐driven topologically associated domains and regulates the expression of HOXA genes crucial for AML differentiation. HOTTIP regulates hematopoietic differentiation by interacting with hematopoietic‐specific epigenetic regulators and transcription factors such as STAT5a, MYC, RUNX1, and DOT1L. They further demonstrated that HOTTIP expression in murine hematopoietic stem cells led to an increased expansion of c‐Kit+ cells and was accompanied by increased self‐renewal capabilities and impaired differentiation (Luo et al., 2019). Likewise, the lncRNA HOXBLINC has recently been identified as a key modulator of myeloid cell differentiation in NPM1‐mutant AML. As an AML‐initiating lesion, NPM1 mutation, and consequent cytoplasmic mis‐localization of NPM1 protein, maintains a distinctive transcriptional signature in AML cells, characterized by upregulation of HOXA and HOXB cluster genes. In a study by Zhu et al., RT‐qPCR analysis determined that HOXBLINC expression was high in HSCs, low in progenitor and negligible in mature myeloid cells further suggesting that HOXBLINC plays a role in myeloid stem cell differentiation. In murine models of NPM1 mutant AML, transgenic expression of HOXBLINC enhanced symmetric self‐renewal of stem cells and reduced the proportion of cells undergoing symmetric differentiation (Zhu et al., 2021).

Another lncRNA that regulates differentiation in NPM1 mutant AML is the lncRNA LONA. LONA is highly expressed in NPM1 mutant AML compared to NPM1 WT AML and interestingly nuclear localization of LONA was inversely correlated to NPM1 protein localization. Gourvest et al. demonstrated that deregulation of LONA impairs myeloid differentiation in an NPM1 mutant‐dependent fashion. Using Vitamin D3‐induced differentiation assays the authors showed that expression of LONA in NPM1 mutant AML correlated with a decrease in the number of cells that underwent differentiation as measured by CD11b expression. This differentiation block was recovered by GapmeR‐induced knockdown of LONA thereby demonstrating that LONA is directly responsible for halting differentiation of AML blasts. Interestingly, LONA expression was found to enhance Vitamin D3‐induced myeloid cell differentiation in NPM1 wild‐type AML cells thereby highlighting the context‐specific roles of lncRNAs (Gourvest et al., 2021).

As mentioned above, lncRNA function is often highly context‐specific. In fact, in some cases a single lncRNA can induce opposing phenotypes in the same disease. For example, the lncRNA HOTAIR, which has well‐defined roles in solid cancers, has been shown to both induce differentiation and maintain self‐renewal of AML through two defined mechanisms. In a 2018 study, Gao et al. found that knockdown of HOTAIR in AML reduced the frequency and self‐renewal capabilities of leukemic stem cells (LSCs) via promoting EZH2‐mediated tri‐methylation of Lys 27 of histone (H3K27me3) of the p15 promoter. This study demonstrated that expression of HOTAIR is required for LSC self‐renewal and maintenance of immature blast production (Gao et al., 2018). In contrast, a recent study from Hu et al reported that HOTAIR expression is vital for the differentiation of AML blasts treated with ATRA. siRNA knockdown of HOTAIR was found to block differentiation and keep AML blasts in an immature state. This process was mediated by HOTAIR‐induced downregulation of miR‐17‐5p and subsequent downregulation of the p21/cyclin D1/CDK4 pathway (Hu et al., 2021). The discrepancy in the role of HOTAIR in AML further highlights both the heterogeneity of AML and lncRNA action as well as our limited knowledge of the complexity of lncRNA function in AML.

Finally, the lncRNA HOXA10‐AS has also been described to perturb myeloid cell differentiation. In the studies previously mentioned, Al‐Kershi et al. described how HOXA10‐AS overexpression significantly impaired HSC differentiation under monocytic culture conditions. Flow cytometry analysis showed a reduction of CD11b+/CD14+ and CD14+/CD163+ monocytic cells in HSCs transduced to express HOXA10‐AS, compared with the empty vector controls. This differentiation block was also verified morphologically; HOXA10‐AS‐expressing cells were seen to resemble immature monocytic progenitors compared with the typical monocytes as characterized by condensed nuclei, less cytoplasmic space and a smaller cell size (Al‐Kershi et al., 2019).

In summary, the regulation of cell differentiation is widely mediated by lncRNA action. Given the success of ATRA‐based therapies in treating APML via inducing blast differentiation, there is a legacy of the value of overcoming differentiation block in the treatment of leukemia. Therefore, further studies into targeting lncRNAs as potential therapeutic targets to induce differentiation in AML blasts could provide major therapeutic benefits.

### 4.3 Therapy resistance

**FIGURE 4:**
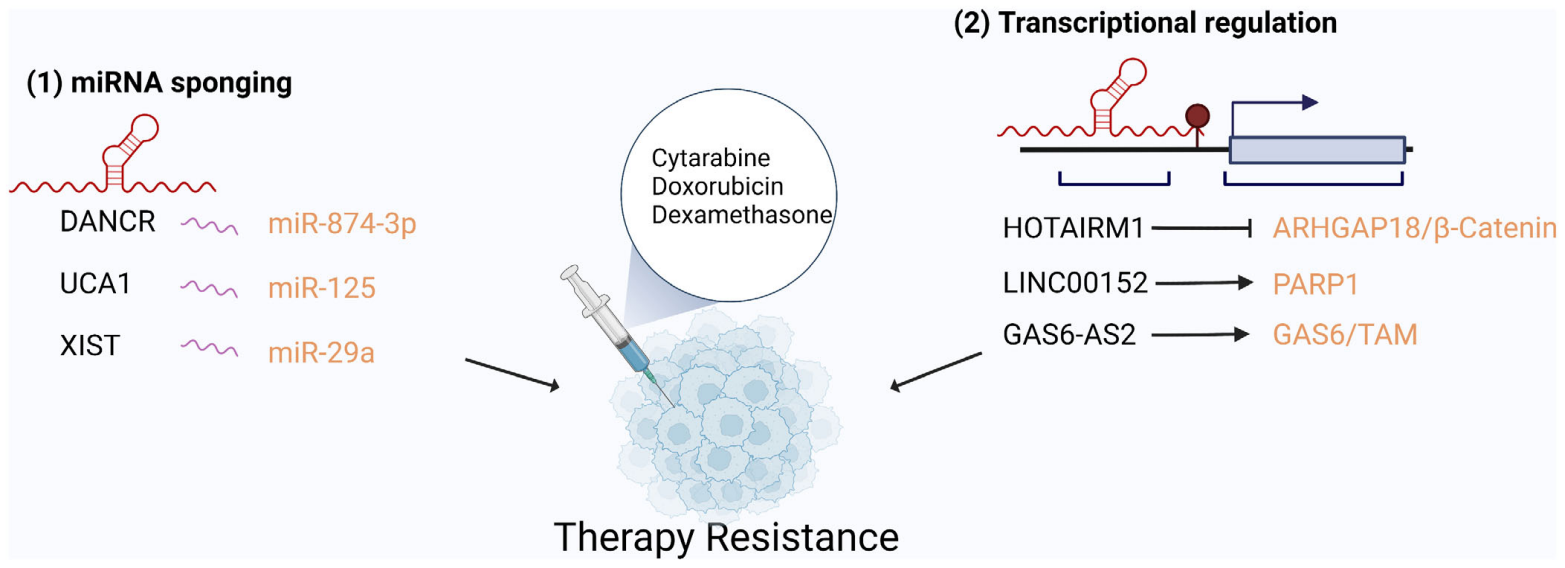
lncRNAs promote drug resistance in AML. lncRNAs promote drug resistance pathways and confer resistance to standard of care therapies of AML through (1) sponging miRNAs to promote resistance gene expression or (2) regulating the transcription of key drug resistance genes.

Resistance to chemotherapy poses a major clinical obstacle to the management of AML. While many patients respond positively to initial treatments, cases of refractory and relapsed AML that develop chemoresistance are a major cause of death (Rashidi et al., 2018). While broad‐spectrum drugs such as Venetoclax have shown moderate success in combination with conventional chemotherapies, these remissions are often short lived (Othman et al., 2021). The exact mechanisms of drug resistance development are currently being investigated and it is likely that many mechanisms such as acquired mutations, increased drug efflux and Darwinian clonal selection work in tandem to drive both relapse and therapy resistance in patients. Given the well‐documented roles of lncRNAs in regulating gene expression and cellular pathways, it is no surprise that several lncRNAs have been found to drive therapy resistance in AML (Figure 4).

For example, the lncRNA DANCR has been identified to confer resistance to the standard of care drug cytarabine (Ara‐C) in AML cell lines. DANCR has been reported to be highly expressed in LSCs, a cellular population that is typically resistant to Ara‐C (Bill et al., 2019). A recent study by Zhang et al. showed that DANCR expression was upregulated in AML cells exposed to Ara‐C in a dose‐dependent manner. Lentiviral vector‐mediated overexpression of DANCR conferred resistance to Ara‐C and this resistance was reversed following siRNA knockdown of DANCR. It was found that DANCR acts as a miRNA sponge to target miR‐874‐3p and thereby relieves repression of the drug resistance gene ATG16L1. Accordingly, knockdown of DANCR was shown to reduce ATG16L1 expression and confer Ara‐C sensitivity to AML cell lines (H. Zhang et al., 2021). Similarly, the lncRNA UCA1 has also been identified to mediate chemotherapy resistance via miRNA sponging. UCA1 was found to be upregulated in AML patient samples following Ara‐C and doxorubicin (Dox) treatment and overexpression of UCA1 conferred Dox resistance to AML cell lines in vitro. In a similar manner to DANCR, UCA1 induced resistance of Dox via sponging miR‐125 and relieved miRNA inhibition of the hypoxia‐induced transcription factor HIF‐1α. This increased HIF‐1α expression increased the glycolytic rate of AML cells and allowed them to resist Dox chemotherapy. As Dox is a commonly used chemotherapeutic agent for the treatment of AML it is no surprise that other lncRNAs have been identified to drive Dox resistance in AML (Y. Zhang et al., 2018). The lncRNA XIST, one of the first lncRNAs described (Loda & Heard, 2019), has also been identified to confer Dox resistance in AML. XIST was found to sponge miR‐29a and thereby relieve repression of the oncogene MYC. Silencing XIST via siRNA knockdown in the AML cell line KG‐1 increased sensitivity to Dox by downregulating MYC and promoting apoptosis (C. Wang et al., 2020). The fact that new roles for XIST, one of the best‐studied lncRNAs, are still being discovered is testimony to the knowledge gap in lncRNAs in AML.

In some cases, a single lncRNA can be responsible for resistance to a variety of drugs via modulating different cellular pathways an example of this is the lncRNA HOTAIRM1 (HOTAIR myeloid 1) which, like the lncRNA HOTAIR, is named after its genomic position antisense and intergenic to human HOX genes. HOTAIRM1 is highly specific for myeloid cells and its expression has been implicated with myeloid cell differentiation and in the development of AML (L. Chen, Hu, et al., 2020; Zhang et al., 2009). HOTAIRM1 has recently been shown to enhance dexamethasone resistance by transcriptionally inhibiting ARHGAP18 which via RHOA/ROCK1 activity increases expression of the antiapoptotic gene BCL2 and decreases the proapoptotic gene BCL2L11 (BIM). Consequently, knockdown of HOTAIRM1 was able to relieve ARHGAP18 repression and re‐sensitize cells to dexamethasone (L. Liang et al., 2021). Alternatively, HOTAIRM1 has also been documented to drive AML resistance to Ara‐C via modulating the Wnt/β‐catenin pathway. In a separate study, knockdown of HOTAIRM1 enhanced cytarabine‐induced apoptosis in AML cell lines, suppressed glycolysis rates and impaired activity of the Wnt/β‐catenin pathway as evidenced by reduced β‐catenin and c‐MYC expression. Furthermore, overexpression of β‐catenin was able to rescue this phenotype and restore resistance to Ara‐C in HOTAIRM1 knockdown cells (L. Chen, Hu, et al., 2020). These data further highlight the many roles that lncRNAs have in drug resistance and suggest that targeting a single lncRNA could have therapeutic value for a variety of chemotherapies.

Multiple studies have suggested that CD34 + CD38− LSCs are a leading cause of chemoresistance in AML patients (Thomas & Majeti, 2017). Therefore, identifying lncRNAs that are specifically expressed in LSCs can provide targets to specifically overcome drug resistance in LSCs and eradicate this rare population. A recent study from Cui et al, identified LINC00152 as a CD34 + CD38− LSC specific lncRNA whose expression was highly correlated with the landmark “LSC17” gene expression signature (Ng et al., 2016), thereby suggesting an important role of LINC00152 in leukemic stemness. The same study demonstrated that knockdown of LINC00152 increased LSC sensitivity to Dox and reduced expression of the DNA damage repair protein PARP1 (Cui et al., 2021). While the exact mechanism of how LINC00152 regulates drug resistance is still unknown, this study serves as an example of the value of targeting lncRNAs to overcome drug resistance in the LSC fraction.

Large‐scale genetic screening of lncRNAs now provides researchers with a high throughput method to identify genes associated with drug resistance and sensitivity. While traditional investigations on the genes responsible for therapy resistance have focused on protein‐coding genes, recent studies are now expanding this methodology to the noncoding aspects of the genome. A recent study from Bester et al. employed a genome‐wide CRISPR screen to identify lncRNAs vital to Ara‐C resistance in the AML cell line MOLM14. Utilizing an extensive CRISPR library that targeted 14,701 lncRNA genes, the authors identified and validated the lncRNA GAS6‐AS2 as vital for resistance to Ara‐C. Follow‐up validation experiments determined that GAS6‐AS2 promotes hyperactivation of the GAS6/TAM pathway, a resistance mechanism in multiple cancers, and thereby confers drug resistance in MOLM14 cells (Bester et al., 2018). With the increased utilization of genome‐wide screens targeting lncRNAs, there is no doubt that many more lncRNAs which drive therapy resistance will be discovered in the near future.

### 4.4 Tumor suppressors

**FIGURE 5:**
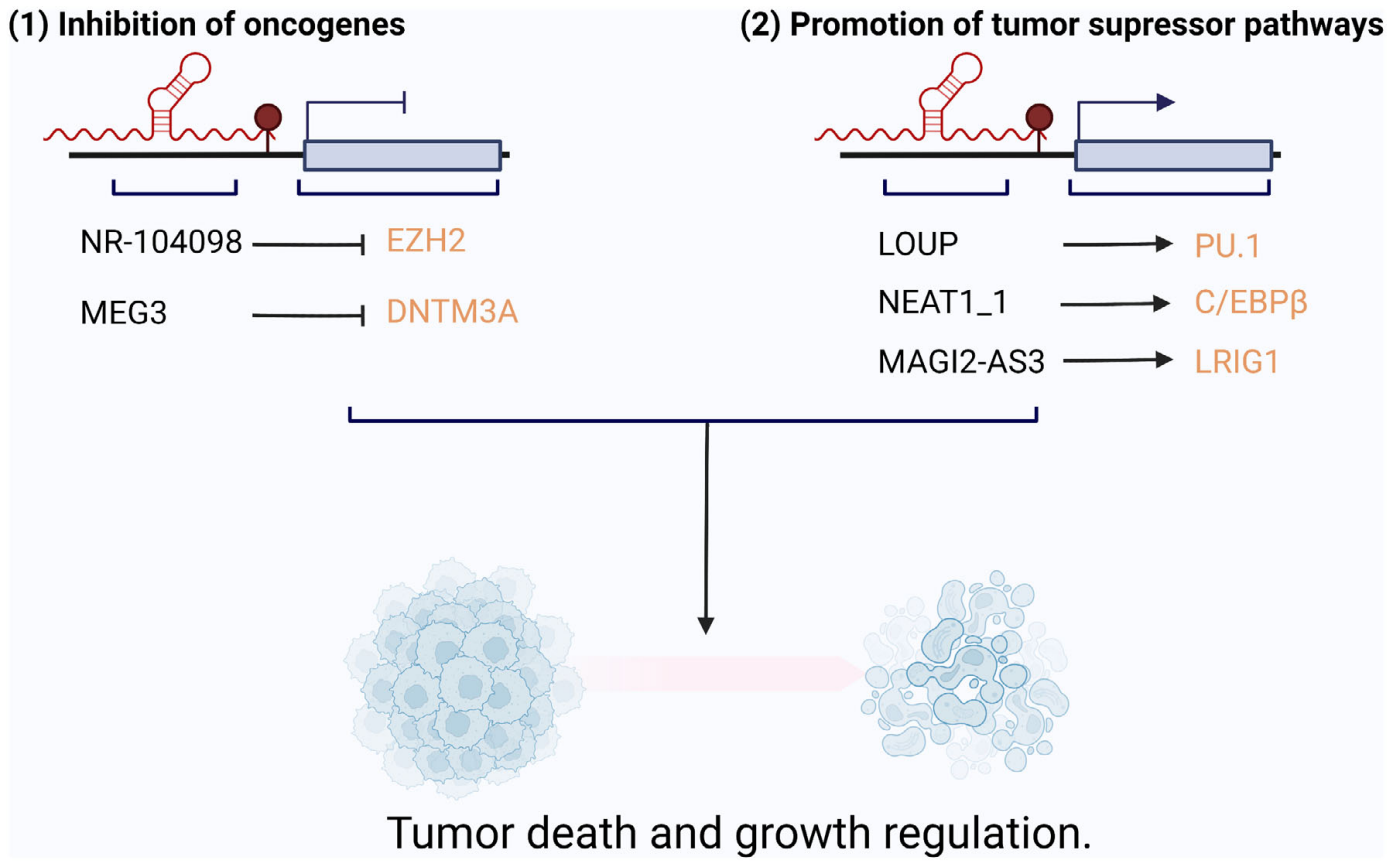
lncRNAs act as tumor suppressors in AML. lncRNAs can serve as tumor suppressors in AML through (1) inhibiting pro‐oncogenic pathways or (2) promoting tumor suppressor pathways and regulating cell growth.

While the oncogenic role of lncRNAs has been discussed above, there are a number of lncRNAs that positively regulate apoptotic pathways, cell cycle arrest and balanced metabolic profiles (Guzel et al., 2020). Like oncogenic lncRNAs, these lncRNAs are often dysregulated in tumors, however, their roles are vital for normal and balanced cell growth. Appropriately, this class of lncRNA are defined as “tumor suppressor lncRNAs.” In the case of AML, a small number of lncRNAs have so far been identified to act as tumor suppressor lncRNAs and found to act through similar mechanisms to oncogenic lncRNAs namely miRNA sponging, transcriptional regulation and epigenetic regulation (Figure 5).

One such tumor suppressor lncRNA is NR‐104098. Microarray analysis performed by Feng et al. discovered that NR‐104098 was downregulated in AML cells following ATRA‐induced differentiation. Overexpression of NR‐104098 decreased cell proliferation and promoted AML blast differentiation as measured by an increase in C11b and CD14 expression. Feng et al determined that NR‐104098 acts as a scaffold for the transcription factor E2F1 and recruits E2F1 to the promoter of the oncogenic transcriptional repressor EZH2. Reduced EZH2 expression was in turn responsible for induced differentiation and decreased proliferation of AML cells (Feng et al., 2020). Another lncRNA that promotes myeloid differentiation is LOUP. LOUP is a myeloid‐specific lncRNA that directly recruits RUNX1 to the transcriptional promoter and enhancer of the myeloid regulator PU.1 to form an active chromatin loop. This coordination of LOUP and RUNX1 results in myeloid differentiation and inhibition of proliferation in myeloid cells. Consequently, CRISPR‐mediated knockout of LOUP inhibited PU.1 expression and blocked differentiation in healthy CD34+ cord blood cells. In the case of AML1‐ETO AML, the RUNX1‐ETO fusion protein limits chromatin accessibility at the LOUP locus and causes inhibition of PU.1 expression, thus halting the tumor suppressor role of LOUP and causing differentiation block and increased proliferation (Trinh et al., 2021).

As self‐renewal of LSCs is vital for AML initiation, lncRNAs that deregulate LSC maintenance have the potential to inhibit the disease. One such case is the lncRNA MAGI2‐AS3 which has been reported to inhibit LSC self‐renewal and act as a tumor suppressor in AML. In a study by Chen et al. MAGI2‐AS3 was found to be significantly downregulated in AML LSCs compared to healthy HSC controls, and overexpression of MAGI2‐AS3 inhibited the colony‐forming and self‐renewal ability of LSCs. The authors found that MAGI2‐AS3 inhibits LSC self‐renewal by promoting TET2‐dependent DNA demethylation of the LRIG1 promoter and that NOD/SCID mice injected with LSCs overexpressing MAGI2‐AS3 had increased survival time and lower LSC levels compared to control LSCs (L. Chen, Fan, et al., 2020). In an interesting case, the lncRNA NEAT1 has also been identified as a tumor suppressor in AML. Despite being upregulated in a variety of human cancer types and having clear oncogenic roles in solid tumors, prognostic analysis of the short isoform NEAT1 (NEAT1_1) revealed that high expression is positively correlated with OS of AML patients. Furthermore, NEAT1_1 expression was downregulated in relapse compared to diagnosis patients and further downregulated in LSCs, suggesting its down‐regulation is associated with stemness. Knockdown of NEAT1_1 in LSCs significantly increased colony formation, enhanced proliferation and suppressed apoptosis. Similarly, NSG mice transplanted with NEAT1_1 knockdown AML patient samples displayed enhanced engraftment and decreased survival which was rescued by restoring NEAT1_1 expression. NEAT1_1 exerts its tumor suppressor effects by binding to the promoter and enhancing transcription of C/EBPβ, an important tumor suppressor and regulator of myeloid differentiation. This in turn causes NEAT1_1 transport to the cytoplasm, where the lncRNA inhibits the key WNT pathway component DVL‐2 and disrupts LSC self‐renewal (Yan et al., 2021).

Finally, the lncRNA MEG3 has also been found to act as a tumor suppressor in AML. Unlike NEAT1 which has both oncogenic and tumor suppressor roles, MEG3 has been well reported to inhibit tumor growth in a p53‐dependant manner and this role is conserved in the case of AML (Zhou et al., 2012). A recent study from Lyu et al. demonstrated that MEG3 expression is downregulated in AML patient samples compared to healthy CD34+ cells. Likewise, NOD‐SCID mice transplanted with AML cell lines overexpressing MEG3 had decreased leukemic infiltration to the spleen and increased survival, demonstrating the tumor suppressor activity of MEG3 in AML. Interestingly, overexpression of MEG3 in both the p53 WT MOLM‐13 and p53 mutant U937 AML cell lines was found to significantly reduce cell proliferation and induce G0/G1 cell cycle arrest and apoptosis, suggesting that MEG3 can function as a tumor suppressor in a p53‐independent pathway. In the same study, the authors determined that MEG3 is transcriptionally regulated by WT1 and TET2, which are commonly inactivated in AML, and that MEG3 directly represses the expression of the oncogene DNTM3A (Lyu et al., 2017).

While a relatively small number of lncRNAs have been identified as tumor suppressor lncRNAs, these studies provide valuable insight into core tumor‐suppressive pathways in AML and their key regulators. This knowledge could be utilized in the clinic to treat patients who have refractory disease or therapy‐resistant relapse.

### 4.5 Biomarkers

**FIGURE 6:**
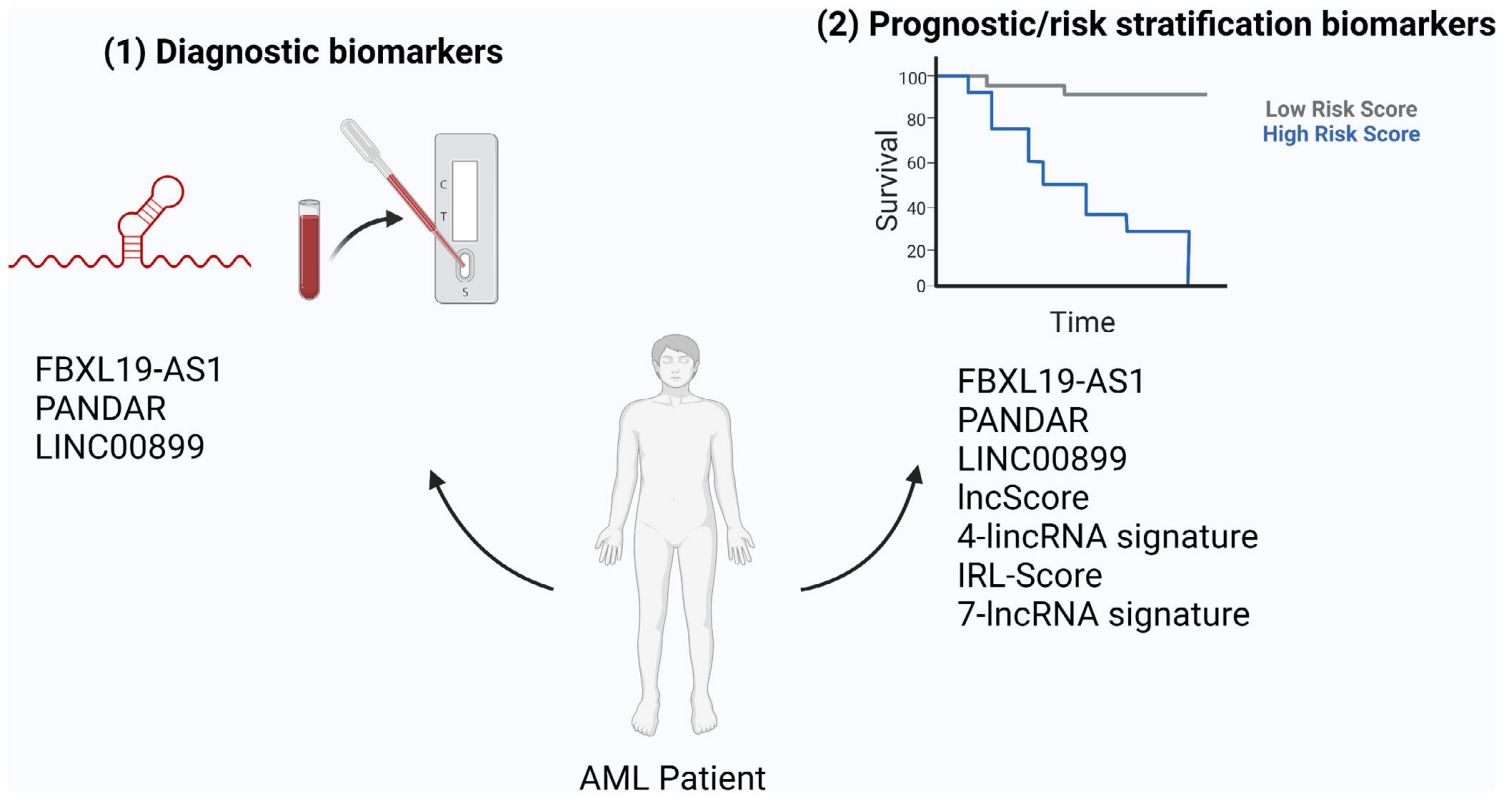
The role of lncRNAs as biomarkers in AML. lncRNAs can act as (1) diagnostic or (2) prognostic biomarkers for patients with AML. lncRNAs can function as biomarkers individually or as part of a lncRNA “signature.”

It is possible that as we investigate many more lncRNAs it will be discovered that a number have no specific mechanisms of action and are by‐products of transcriptional events. However, lncRNAs that do not regulate cellular processes can still have valuable therapeutic potential in the way of acting as biomarkers for the diagnosis and risk stratification of cancer. The first lncRNA approved by the FDA for diagnostic purposes in prostate cancer, PCA3, is a promising first step in the use and exploration of lncRNAs to be utilized similarly in AML (McKiernan et al., 2016). Recent studies have identified a small number of lncRNAs as diagnostic or prognostic biomarkers in AML (Figure 6).

For example, overexpression of the lncRNA PANDAR has been associated with adverse prognosis in AML. PANDAR is highly expressed in AML patients compared to healthy controls and receiver operating characteristic (ROC) curve analysis demonstrated that PANDAR expression could be used as a diagnostic biomarker to distinguish AML patients from healthy controls. Furthermore, high PANDAR expression was associated with a higher blast count at diagnosis and logistic regression analysis revealed that PANDAR expression was an independent risk factor that affected chemotherapy response with high expressors more likely to be unresponsive to therapy (Yang et al., 2018). Likewise, high expression of PANDAR was associated with lower OS time compared to low PANDAR expressing patients, thereby demonstrating the prognostic value of PANDAR as a biomarker. Similarly, the lncRNA FBXL19‐AS1 has recently been identified as a novel biomarker for pediatric patients with AML. ROC curve analysis of healthy controls and pediatric AML patients revealed that serum levels of FBXL19‐AS1 was able to distinguish AML patients from healthy controls and therefore FBXL19‐AS1 is a potential diagnostic marker for AML patients. Interestingly, FBXL19‐AS1 expression was also correlated with unfavorable cytogenetic groups and poor survival, again highlighting the potential of FBXL19‐AS1 as a prognostic biomarker (Sheng et al., 2021). Finally, the lncRNA LINC00899 was also found to be upregulated in serum and bone marrow of AML patients compared to healthy controls. Moreover, ROC curve analyses showed that serum levels of LINC00899 could differentiate AML patients from healthy controls, highlighting the diagnostic potential of LINC00899 as a biomarker. Much like PANDAR and FBXL19‐AS1, LINC00899 expression was positively correlated with shorter OS and Cox proportional hazards analysis showed that high levels of LINC00899 was an independent prognostic marker of poor outcome (Y. Wang et al., 2018).

In contrast to the use of individual lncRNAs as biomarkers, several groups have investigated the value of lncRNA expression profiles in predicting disease characteristics and outcomes. A study by Smith et al., investigated the power of lncRNA expression profiles in pediatric AML at predicting outcome without knowledge of somatic variants, thus creating a variant‐agnostic prognostic indicator. Using a machine‐learning‐based training model, the authors identified a set of 37 lncRNAs whose expression was associated with patient outcome. Using this cohort of lncRNAs they developed a prognostic scoring system dubbed “lncScore.” In their analysis, those with positive lncScores had a lower overall and event‐free survival at 5‐years from diagnosis compared to those with negative scores. They also determined that lncScore was an independent prognostic factor from cytogenetic and molecular risk grouping, suggesting the lncRNA signature may provide additional prognostic information over traditional risk stratification methods (Farrar et al., 2023). Similarly, a study by Beck et al. identified a subset of four long intergenic noncoding RNAs (lincRNAs) that had prognostic value across three independent patient cohorts in a multivariable analysis that included age, gender, and existing European Leukemia Net (ELN) risk stratification. The authors demonstrated that using this signature, patients in each ELN risk group could be further split into good or poor prognosis cohorts thereby potentially providing clinical management insights for intermediate‐risk groups where treatment strategy is often difficult to determine (Beck et al., 2017).

In a separate study, immune‐related lncRNAs were identified using the TCGA database and a subset of four prognostic lncRNAs were identified using multiple stepwise Cox regression. With this signature of four immune‐related lncRNAs (IRL‐score) the authors were able to accurately predict the OS of AML patients and demonstrated that patients with a high IRL‐score had higher mortality rates. Subsequent stratification analysis demonstrated that IRL‐score could be applied to different clinicopathological subgroups and maintained its predictive power, with the exception of senior patients >65 years old. Finally, IRL‐score was able to predict prognosis independently of blast count and ELN risk stratification system, further demonstrating the prognostic potential of lncRNA signature in AML (R. Li et al., 2022). Comparably, Liu et al. recently identified a prognostic 7‐lncRNA signature for AML which consisted of lncRNAs identified as highly expressed in AML in the TARGET dataset. They found that their 7‐lncRNA signature (LINC00461, RP11—309 M23.1, AC016735.2, RP11—61I13.3, KIAA0087, RORB—AS1, AC012354.6) was able to accurately predict patient outcome with patients with a high “risk score” having poor OS compared to the low‐risk score cohort. Interestingly, none of these lncRNA have defined functions in cancer or AML, thereby highlighting the value of lncRNAs as biomarkers independent of their mechanism (C. Y. Liu et al., 2021).

While a limited number of studies have investigated the use of lncRNAs as biomarkers in AML, the initial results are promising and continued study in this field will likely identify many more predictive lncRNAs.

## 5 LIMITATIONS OF TARGETING lncRNAs IN AML

While the examples provided in this review shine a promising light on the therapeutic potential of lncRNAs in AML as both molecular targets and diagnostic or prognostic biomarkers, there are many limitations to targeting lncRNAs in a clinical setting that remain to be overcome. For example, a major limitation in targeting oncogenic lncRNAs is realizing efficient ways to inhibit lncRNA expression and function. As lncRNAs are classically considered “undruggable” targets, gene silencing methods such as siRNA or anti‐sense oligonucleotides have been employed to perturb target lncRNA expression in research settings (Winkle et al., 2021). However, these methods often fall short in translation due to hurdles in efficient delivery of inhibitory molecules. One promising delivery mechanism is the use of lipid nanoparticles (LNPs). LNPs have shown promise in the delivery of RNA therapeutics such as the COVID‐19 mRNA vaccines (Hou et al., 2021) and this approach has shown success in early in vitro models of AML (Connerty et al., 2021), but the feasibility of LNP‐delivered therapeutics to target lncRNAs in more clinically relevant settings of AML remains to be seen. In addition to LNP‐based therapeutics, small molecule screens to identify compounds that disrupt lncRNA‐protein interaction have been developed to identify potential therapeutic molecules to target lncRNAs (Pedram Fatemi et al., 2015). While this technique presents a promising method to find targeted drugs for disrupting these “undruggable” lncRNAs there has been little published research on these methods in the context of AML.

Furthermore, the fact that lncRNAs are poorly conserved across species represents another limitation in testing efficient lncRNA‐based therapies in AML. Due to the complex roles of lncRNAs, they should be evaluated in appropriate contexts to fully understand the interactions between lncRNA and target genes and proteins. However, lncRNAs are poorly conserved across species and common animal models, such as murine models of cancer, may not be suitable for the study of lncRNA targeting therapeutics as many human lncRNAs are not expressed in mice (Ghanam et al., 2022). Producing transgenic mice models and establishing patient‐derived xenograft models of AML has proven challenging and these limitations pose a hurdle to overcome in the preclinical testing of lncRNAs as treatment targets (Diaz de la Guardia et al., 2021; Ghanam et al., 2022).

Finally, the low abundance of lncRNAs in relation to protein‐coding genes represents another limitation in both their targeting and use as biomarkers in AML (Mattick et al., 2023; Winkle et al., 2021). As alternatively spliced lncRNAs can have starkly different cellular functions, diagnostic, or prognostic tests utilizing lncRNAs must identify the most functionally relevant splicing variants for targeting (Khan et al., 2021). While this represents a hurdle for the clinical use of lncRNAs, recent advances in the analysis of RNA‐seq data and RNA‐seq based alternative splicing analysis have the potential to overcome this issue in the future (Halperin et al., 2021).

These limitations represent a few of the major challenges researchers will need to overcome to exploit the full benefits of targeting lncRNAs in AML. They also highlight the need for continued research in this field.

## 6 CONCLUSION

In conclusion, lncRNAs are key genetic molecules that are major regulators of many cellular processes in AML. We are now appreciating the importance of noncoding RNAs and their ability to not only drive oncogenic processes such as proliferation, differentiation block, and therapy resistance, but to also act as tumor suppressors which are critical to stop the malignant transformation of healthy cells. Furthermore, the context‐specific expression of lncRNAs enables them to be used as biomarkers for the diagnosis and risk stratification of AML. In the case of deadly cancers like AML, where survival rates have reached a plateau and new therapeutic targets and methods of risk stratification are required, lncRNAs have major potential to overcome the shortcomings of traditional protein‐centric views of cancer biology and better serve clinicians and researchers with an improved understanding of AML biology and disease treatment. While this review is far from an exhaustive list of lncRNAs identified to play a role in AML, the studies highlighted in this article exemplify the limited understanding we have of lncRNAs in AML and demonstrate the need for future studies to uncover more lncRNAs which are key molecular drivers and promising therapeutic targets in AML.

## AUTHOR CONTRIBUTIONS

**Patrick Connerty:** Conceptualization (lead); writing – original draft (lead); writing – review and editing (equal). **Richard B. Lock:** Writing – review and editing (equal).

## FUNDING INFORMATION

This work was funded by Anthony Rothe Memorial Trust Grant (RG213331) to Patrick Connerty, as well as the National Health and Medical Research Council of Australia (NHMRC Fellowship APP1157871 to Richard B. Lock).

## CONFLICT OF INTEREST STATEMENT

The authors declare no conflict of interest.

## RELATED WIREs ARTICLES

Micro‐RNAs: A safety net to protect hematopoietic stem cell self‐renewal

Noncoding RNAs in oral cancer

## Data Availability

Data sharing is not applicable to this article as no new data were created or analyzed in this study.
